# Bcl‐xL expression following articular cartilage injury and its effects on the biological function of chondrocytes

**DOI:** 10.1002/elsc.202000039

**Published:** 2020-10-13

**Authors:** Zhengjun Pan, Hao Yin, Shuangli Wang, Gaoxin Xiong, Zongsheng Yin

**Affiliations:** ^1^ Orthopedics Department The First Affiliated Hospital of Anhui Medical University Hefei Anhui P. R. China; ^2^ Orthopedics Department The First People's Hospital of Hefei Hefei Anhui P. R. China

**Keywords:** Bcl‐xL, BMP, cartilage damage, caspase‐3, CKLF1, IGF‐1, IL‐1β, MMP‐3, osteoarthritis

## Abstract

This study aimed to investigate the expression of B‐cell lymphoma‐extra large (Bcl‐xL) in cartilage tissues following articular cartilage injury and to determine its effects on the biological function of chondrocytes. A total of 25 necrotic cartilage tissue samples and 25 normal tissue samples were collected from patients diagnosed with osteoarthritis at our hospital from December 2015 to December 2018. The mRNA expression levels of Bcl‐xL, caspase‐3, and matrix metalloproteinase‐3 (MMP‐3) in the normal and necrotic tissues were examined via quantitative polymerase chain reaction, and their protein expression levels were detected via western blotting. The expression levels of Bcl‐xL, insulin‐like growth factor‐1 (IGF‐1), and bone morphogenetic protein (BMP) were significantly lower but those of caspase‐3, MMP‐3, interleukin‐1β (IL‐1β), and chemokine‐like factor 1 (CKLF1) levels were markedly higher in necrotic cartilage tissues than in normal tissues. Following cell transfection, the expression levels of Bcl‐xL, IGF‐1, and BMP were remarkably higher but those of caspase‐3, MMP‐3, IL‐1β, and CKLF1 were notably lower in the Si‐Bcl‐xL group than in the NC group. The Si‐Bcl‐xL group showed significantly lower cell growth and noticeably higher apoptosis rate than the NC group (normal control group). The expression of Bcl‐xL is reduced following articular cartilage injury, and this reduction promotes the proliferation and inhibits the apoptosis of chondrocytes. Therefore, Bcl‐xL could serve as a relevant molecular target in the clinical practice of osteoarthritis and other diseases causing cartilage damage.

**Abbreviations**
Bcl‐xL: B‐cell lymphoma‐extra large
BMP: bone morphogenetic protein
CKLF1: chemokine‐like factor 1
IGF‐1: insulin‐like growth factor‐1
IL‐1β: interleukin‐1β; NC group, normal control group

## 1 INTRODUCTION

Cartilage damage occurs in orthopedic diseases such as osteoarthritis, femoral injury, and osteochondral defect of the knee [1, 2, 3]. Among these, osteoarthritis is an inflammatory disease in which the risk of cartilage damage to the lateral femoral joint is increased due to reduced strength of the quadriceps. Inflammation occurs with this damage, accompanied by monocyte and proinflammatory mediators as well as many other upregulated factors [4, 5, 6]. Therefore, related clinical studies can formulate corresponding molecular‐targeted treatment plans by identifying corresponding factors, such as molecular markers [7]. The antiapoptotic protein B‐cell lymphoma‐extra large (Bcl‐xL) is one such molecular marker [8].

Bcl‐xL, as a member of the Bcl‐2 family, exhibits antiapoptotic effects and is therefore highly expressed as a promoting factor in various types of cancers [9, 10]. However, in many studies, including those on osteoarthritis, Bcl‐xL has been shown to exhibit certain anti‐inflammatory activities. In cartilage damage caused by osteoarthritis, the higher the apoptosis, the more severe the damage; therefore, the antiapoptotic effects of Bcl‐xL on cells alleviate the inflammatory response to some extent [11, 12, 13, 14]. In a rabbit model of cartilage damage, Bcl‐xL overexpression in HUCSCs prevented apoptosis induced by chondrogenic differentiation in vitro, and Bcl‐xL gene modifications reduced apoptosis induced by chondrogenic differentiation and improved the efficiency of cartilage damage repair. Moreover, Bcl‐xL is related to caspase‐3 and matrix metalloproteinase‐3 (MMP‐3) expression [15]. Experiments in a rat model of knee osteoarthritis have demonstrated that increased caspase‐3 and MMP‐3 expression results in increased apoptosis of chondrocyte, whereas decreased caspase‐3 and MMP‐3 expression results in significantly improved symptoms of knee osteoarthritis [16]. However, there are limited data on the association of human Bcl‐xL with caspase‐3 and MMP‐3 and its role in human chondrocytes. Therefore, the purpose of this study was to analyze Bcl‐xL expression following articular cartilage injury and to determine its effect on the biological functions of human chondrocytes.

## 2 MATERIALS AND METHODS

### 2.1 Data collection

We collected 25 samples of necrotic cartilage tissues from patients diagnosed with osteoarthritis at our hospital as well as 25 samples of normal tissues from December 2015 to December 2018. Inclusion criteria were as follows: patients aged >18 years who were diagnosed with osteoarthritis at our hospital and without other inflammations that can impact our study results or any major blood disorders. Exclusion criteria were as follows: patients with hemorrhagic shock caused by trauma, those who had not received surgical treatment, or those with other serious life‐threatening diseases. Patients and their families signed an informed consent form. This research was approved by the IRB of the authors’ affiliated institutions.

### 2.2 Main reagents and detection methods

#### 2.2.1 Main reagents

Human normal cartilage C28/I2 cells were purchased from Shanghai YS Industrial Co., Ltd. Dulbecco's modified Eagle medium (DMEM) was obtained from Hunan Fenghui Biotechnology Co., Ltd. The cell cycle detection kits were obtained from Shanghai ML Biotechnology Co., Ltd. The apoptosis detection kit and transfection reagent Lipofectamine TM3000 were purchased from Sigma‐Aldrich (Shanghai) Trading Co., Ltd. TRIzol reagent was purchased from Shanghai Yuanye Biotechnology Co., Ltd. Primer sequences and transfection plasmids of Bcl‐xL, caspase‐3, MMP‐3, and internal controls were synthesized and designed by Shanghai Xinghan Biotechnology Co., Ltd (Table 1).

**PRACTICAL APPLICATION**

This study investigates Bcl‐xL expression in cartilage tissues following articular cartilage injury and its effects on the biological function of chondrocytes. The results show that Bcl‐xL expression is reduced following articular cartilage injury, thereby promoting the proliferation and inhibiting the apoptosis of chondrocytes. Bcl‐xL can serve as a relevant molecular target in the clinical practice of osteoarthritis and other diseases causing cartilage damage.

**TABLE 1:** Sequences of the related primers

Factors	Upstream primer	Downstream primer
Bcl‐xL	5′‐ATGTCTCAGAGCAACCGGGAGCTG‐3′	5′‐GTCATTTCCGACTGAAGAGTGAGCCC‐3′
Caspase‐3	5′‐TGTGGCATTGAGACAGAC‐3′	5′‐CACTTGCCATACAAACTA‐3′
MMP‐3	5′‐TGTCCCGTTTCCATCTCTCT‐3′	5′‐ATCAAACCTCCAGTATTTGT‐3′
β‐actin	5′‐ATACGCTGGGATGAGCACTGG‐3′	5′‐TCTTTGCGGATGTCCACGTC‐3′

Bcl‐xL, B‐cell lymphoma‐extra large; MMP‐3, matrix metalloproteinase‐3.

### 2.3 Detection of the mRNA expression levels of Bcl‐xL, caspase‐3, and MMP‐3

The mRNA expression levels of Bcl‐xL, caspase‐3, and MMP‐3 in normal and necrotic cartilage tissues as well as in the transfected chondrocytes were detected via quantitative polymerase chain reaction (qPCR). First, total RNA was extracted. Then, qPCR was performed using the US ABI7500 Real‐Time PCR instrument with the primers designed by Shanghai Sangon Biotechnology Co., Ltd. The sequences of the upstream and downstream primers are listed in Table 1. Experimental results were analyzed using relative quantitative methods.

### 2.4 Detection of the protein expression levels of Bcl‐xL, caspase‐3, and MMP‐3

The protein expression levels of Bcl‐xL, caspase‐3, and MMP‐3 in serum samples from the two groups were detected via western blotting. The ratios of Bcl‐xL/GAPDH, caspase‐3/GAPDH, and MMP‐3/GAPDH represent relative expression levels.

### 2.5 Detection of related cytokine levels

An enzyme‐linked immunosorbent assay kit purchased from Suzhou Elisa Biotechnology Co., Ltd. was used to measure the levels of related cytokines—interleukin‐1β (IL‐1β), insulin‐like growth factor‐1 (IGF‐1), chemokine‐like factor 1 (CKLF1), and bone morphogenetic protein (BMP)—in cartilage tissues and transfected cells.

### 2.6 Cell culture and transfection

C28/I2 cells were routinely subcultured in high‐glucose DMEM containing 10% fetal bovine serum in a cell culture incubator at 37°C and with 5% CO_2_. Before transfection, cells were seeded into 24‐well plates and were stimulated with the serum of patients with sepsis to simulate blood vessels after sepsis. Then, the cells were divided into two groups: Si‐Bcl‐xL and NC. The cells were transfected using the Lipofectamine TM3000 kit, and the expression levels of Bcl‐xL, caspase‐3, and MMP‐3 in both groups were detected.

### 2.7 Detection of cell proliferation

The two groups of transfected C28/I2 cells were inoculated in 96‐well plates with three replicates in each well. At 24, 48, and 72 h, 20 μL of cell MTS Cell Proliferation Colorimetric Assay reagent was added to each well 2 h before the end of the culture. After 2 h, cell proliferation was assessed using an automatic enzyme microplate reader at 490 nm.

### 2.8 Detection of apoptosis

The apoptosis of C28/I2 cells was detected using an apoptosis detection kit. Cells were transfected for 72 h and then stained with annexin V/propidium iodide in a 6‐well plate, followed by detection using the Coulter CytoFLEX flow cytometer purchased from Beckman Coulter (USA). The experiment was repeated three times.

### 2.9 Statistical analysis

SPSS19.0 was used for comprehensive statistical analysis of the data. Measurement data (mRNA and protein expression levels of Bcl‐xL, caspase‐3, MMP‐3, IL‐4, IL‐1β7, and IL‐33 in transfected cells and in bone tissues as well as cell proliferation and apoptosis rates) were expressed as X ± S and verified using the *t*‐test. The least significant difference method was used for post‐hoc analysis. A statistically significant difference was assumed at *p* < 0.05.

## 3 RESULTS

### 3.1 Relative expression levels of Bcl‐xL in normal and necrotic cartilage tissues

The mRNA expression levels of Bcl‐xL in normal and necrotic cartilage tissues were 4.54 ± 0.78 and 1.45 ± 0.23, respectively, and the corresponding protein expression levels were 3.21 ± 0.62 and 1.52 ± 0.15, respectively. The results show that the relative expression levels of Bcl‐xL are higher in normal cartilage tissues than in necrotic cartilage tissues (*p* < 0.05) (Table 2).

**TABLE 2:** Expression of Bcl‐xL in necrotic and normal cartilage tissues (n = 25)

Factors	Normal cartilage tissues	Necrotic cartilage tissues	t	*p*
mRNA	4.54 ± 0.78	1.45 ± 0.23	19.00	<0.001
Protein	3.21 ± 0.62	1.52 ± 0.15	13.25	<0.001

Bcl‐xL, B‐cell lymphoma‐extra large.

### 3.2 Expression levels of caspase‐3 and MMP‐3 in normal and necrotic cartilage tissues

**FIGURE 1:**
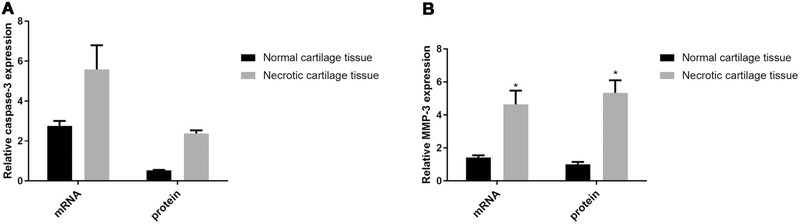
(A) Relative caspase‐3 expression levels in normal and necrotic cartilage tissues. Relative caspase‐3 mRNA and protein expression levels were significantly higher in in necrotic tissues than in normal cartilage tissues (*p* < 0.05). (B) Relative matrix metalloproteinase‐3 (MMP‐3) expression levels in normal and necrotic cartilage tissues. Relative MMP‐3 mRNA and protein expression levels were significantly higher in in necrotic tissues than in normal cartilage tissues (*p* < 0.05). Note: * indicates *p* < 0.05 compared with normal cartilage tissues

The relative mRNA expression levels of caspase‐3 in normal and necrotic cartilage tissues were 2.76 ± 0.24 and 5.58 ± 1.21, respectively, and the corresponding protein expression levels were 0.53 ± 0.02 and 2.37 ± 0.16, respectively. The relative mRNA and protein expression levels of caspase‐3 were dramatically higher in in necrotic tissues than in the normal cartilage tissues (*p* < 0.05). The relative mRNA expression levels of MMP‐3 in normal and necrotic cartilage tissues were 1.42 ± 0.13 and 4.64 ± 0.83, respectively, and the corresponding relative protein expression levels were 1.01 ± 0.14 and 5.34 ± 0.76, respectively. The above data show that the relative mRNA and protein expression levels of MMP‐3 are markedly higher in necrotic tissues than in normal cartilage tissues (*p* < 0.05) (Figure 1).

### 3.3 Expression levels of IL‐1 β, IGF‐1, CKLF1, and BMP in normal and necrotic cartilage tissues

**FIGURE 2:**
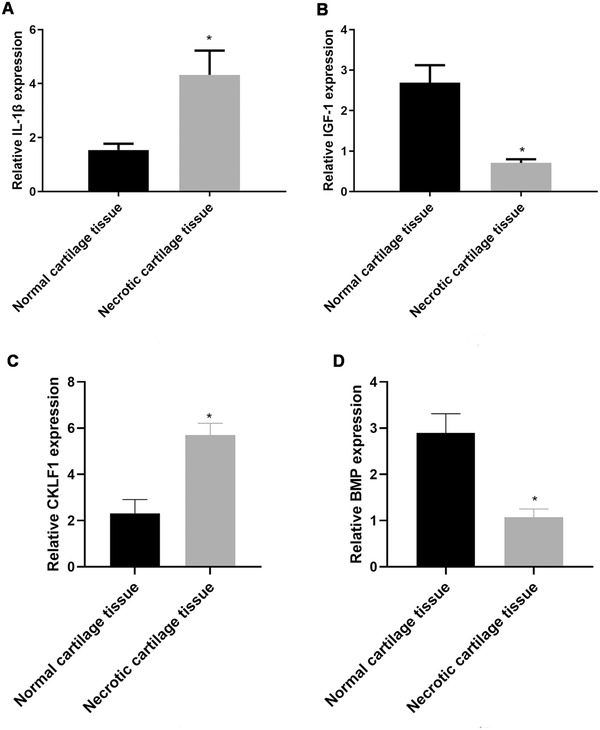
Interleukin‐1β (IL‐1β) expression levels in normal and necrotic cartilage tissues. Relative IL‐1β expression levels were significantly higher in necrotic tissues than that in normal tissues (*p* < 0.05). Insulin‐like growth factor‐1 (IGF‐1) expression levels in normal and necrotic cartilage tissues. Relative IGF‐1 expression levels were significantly lower in necrotic tissues than in normal tissues (*p* < 0.05). Chemokine‐like factor 1 (CKLF1) expression levels in normal and necrotic cartilage tissues. Relative CKLF1 expression levels were significantly higher in necrotic tissues than in normal tissues (*p* < 0.05). Bone morphogenetic protein (BMP) expression levels in normal and necrotic cartilage tissues. Relative BMP expression levels were significantly higher in necrotic tissues than in normal tissues (*p* < 0.05). Note: *indicates *p* < 0.05 compared with normal cartilage tissues

The expression levels of IL‐1β in normal and necrotic cartilage tissues were 1.54 ± 0.23 and 4.32 ± 0.90, respectively; those of IGF‐1 were 2.69 ± 0.43 and 0.71 ± 0.09, respectively; those of CKLF1 were 2.32 ± 0.59 and 5.70 ± 0.51, respectively; and those of BMP were 2.90 ± 0.41 and 1.07 ± 0.18, respectively. These data indicate that the relative expression levels of IL‐1β and CKLF1 are higher but those of IGF‐1 and BMP are lower in necrotic cartilage tissues than in normal cartilage tissues (*p* < 0.05) (Figure 2).

### 3.4 Relative expression levels of Bcl‐xL in transfected cells

The mRNA expression levels of Bcl‐xL in the Si‐Bcl‐xL and NC groups were 1.02 ± 0.13 and 3.74 ± 0.56, respectively, and the corresponding protein expression levels were 0.41 ± 0.07 and 3.27 ± 0.61, respectively. These results suggest that the relative Bcl‐xL mRNA and protein expression levels in the Si‐Bcl‐xL group are lower than that in the NC group (*p* < 0.05) (Table 3).

**TABLE 3:** Relative expression levels of Bcl‐xL in the cells of each group after transfection (n = 25)

Factors	Si‐Bcl‐xL group	NC group	t	*p*
mRNA	1.02 ± 0.13	3.74 ± 0.56	18.20	<0.001
Protein	0.41 ± 0.07	3.27 ± 0.61	21.65	<0.001

Bcl‐xL, B‐cell lymphoma‐extra large.

### 3.5 Effects of Bcl‐xL knockdown on the expression levels of caspase‐3 and MMP‐3

**FIGURE 3:**
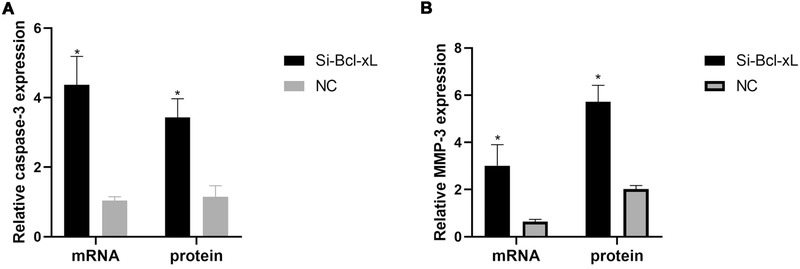
Caspase‐3 expression levels in the Si‐Bcl‐xL and NC groups. Relative caspase‐3 mRNA and protein expression levels were significantly higher in the Si‐Bcl‐xL group than in the NC group (*p* < 0.05). Matrix metalloproteinase‐3 (MMP‐3) expression levels in the Si‐Bcl‐xL and NC groups. Relative MMP‐3 mRNA and protein expression levels were markedly higher in the Si‐Bcl‐xL group than in the NC group (*p* < 0.05). Note: *indicates *p* < 0.05 compared with the NC group. Bcl‐xL, B‐cell lymphoma‐extra large

The mRNA expression levels of caspase‐3 in the Si‐Bcl‐xL and NC groups were 4.37 ± 0.82 and 1.04 ± 0.11, respectively, and the relative protein expression levels were 3.43 ± 0.54 and 1.15 ± 0.32, respectively. Relative mRNA and protein expression levels of caspase‐3 were higher in the Si‐Bcl‐xL group than in the NC group (*p* < 0.05). The mRNA expression levels of MMP‐3 in the Si‐Bcl‐xL and NC groups were 3.01 ± 0.89 and 0.64 ± 0.10, respectively, and the protein expression levels were 5.72 ± 0.70 and 2.02 ± 0.15, respectively. The relative mRNA and protein expression levels of MMP‐3 were higher in the Si‐Bcl‐xL group than in the NC group (*p* < 0.05) (Figure 3).

### 3.6 Effect of Bcl‐xL knockdown on the expression levels of IL‐1β, CKLF1, IGF‐1, and BMP

**FIGURE 4:**
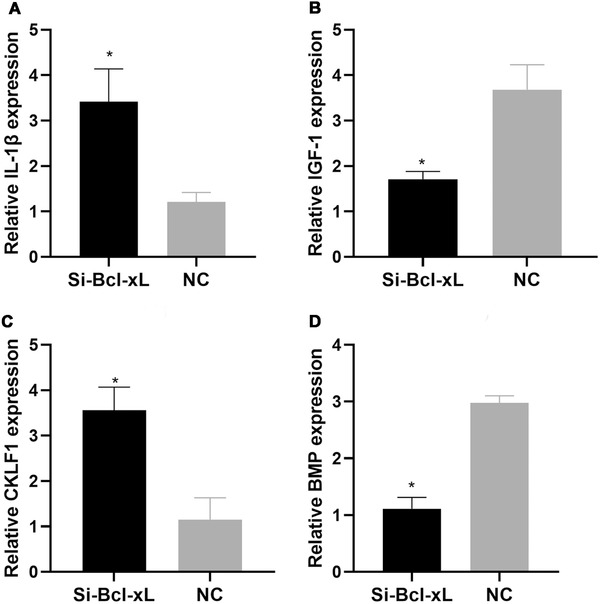
Interleukin‐1β (IL‐1β) expression levels in the Si‐Bcl‐xL and NC groups. Relative IL‐1β expression levels were significantly higher in the Si‐Bcl‐xL group than in the NC group (*p* < 0.05). Insulin‐like growth factor‐1 (IGF‐1) expression levels in the Si‐Bcl‐xL and NC groups. Relative IGF‐1 expression levels were markedly lower in the Si‐Bcl‐xL group than in the NC group (*p* < 0.05). Chemokine‐like factor 1 (CKLF1) expression levels in the Si‐Bcl‐xL and NC groups. Relative CKLF1 expression levels were remarkably higher in the Si‐Bcl‐xL group than in the NC group (*p* < 0.05). Bone morphogenetic protein (BMP) expression levels in the Si‐Bcl‐xL and NC groups. Relative BMP expression levels were dramatically higher in the Si‐Bcl‐xL group than in the NC group (*p* < 0.05). Note: *indicates *p* < 0.05 compared with the NC group. Bcl‐xL, B‐cell lymphoma‐extra large

The expression levels of IL‐1β in the Si‐Bcl‐xL and NC groups were 3.42 ± 0.72 and 1.21 ± 0.21, respectively; those of IGF‐1 were 1.71 ± 0.17 and 3.68 ± 0.55, respectively; those of CKLF1 were 3.56 ± 0.51 and 1.15 ± 0.48, respectively; and those of BMP were 1.11 ± 0.20 and 2.98 ± 0.12, respectively. The relative expression levels of IL‐1β and CKLF1 were higher but those of IGF‐1 and BMP were lower (*p* < 0.05) in the Si‐Bcl‐xL group than in the NC group (Figure 4).

### 3.7 Effect of Bcl‐xL knockdown on C28/I2 cell growth

The growth of C28/I2 cells in Si‐Bcl‐xL group was slower than that of the cells in the NC group from 48 to 72 h. Intragroup comparison revealed that there was a significant difference in the growth of C28/I2 cells between the two groups from 24 to 72 h (*p* < 0.05) (Table 4).

**TABLE 4:** Growth of human normal cartilage C28/I2 cells in the two groups (n = 25)

Time point	Si‐Bcl‐xL group	NC group	T	*p*
24 h	0.49 ± 0.07	0.51 ± 0.07	1.01	0.318
48 h	0.89 ± 0.10*	1.47 ± 0.12*	18.57	<0.001
72 h	1.45 ± 0.15*#	2.80 ± 0.31*#	19.60	<0.001

Bcl‐xL, B‐cell lymphoma‐extra large.

^*^Indicates *p* < 0.05 compared with the growth of C28/I2 cells at 24 h.

^#*^Indicates *p* < 0.05 compared with the growth of C28/I2 cells at 48 h.

### 3.8 Effect of Bcl‐xL knockdown on C28/I2 cells apoptosis

The apoptotic rate of C28/I2 cells in Si‐Bcl‐xL group was 26.34 ± 2.12%, which was higher than that of the cells in the NC group (4.63 ± 0.40%) (*p* < 0.05) (Table 5).

**TABLE 5:** Apoptosis of human normal cartilage C28/I2 cell lines in the two groups

Groups	Si‐Bcl‐xL group	NC group	t	*P*
Apoptosis rate (%)	26.34 ± 2.12	4.35 ± 0.36	50.32	<0.001

Bcl‐xL, B‐cell lymphoma‐extra large.

## 4 DISCUSSION

Although Bcl‐xL serves as a promoter of various types of cancers due to its antiapoptotic effects, requiring targeted knockdown therapy [17, 18, 19], it has also been found to inhibit inflammation in some diseases due to its antiapoptotic effects [20, 21]. The purpose of this study was to investigate the expression of Bcl‐xL in cartilage damage caused by osteoarthritis and determine its effect on chondrocytes.

We first determined the expression levels of Bcl‐xL, caspase‐3, and MMP‐3 in normal and necrotic cartilage tissues. The results revealed that the expression levels of Bcl‐xL were significantly lower but those of caspase‐3 and MMP‐3 were notably higher in necrotic cartilage tissues than in normal cartilage tissues. Caspase‐3 plays an important role in facilitating cell apoptosis, whereas MMP‐3 and inflammatory cytokines promote further deterioration of osteoarthritis and cartilage damage by degrading extracellular matrix components and are highly expressed in inflammatory tissues [22, 23]. The trends of the mRNA and protein expression levels of caspase‐3 and MMP‐3 in normal and necrotic cartilage tissues justify the above point of view. Meanwhile, by comparing the expression levels of Bcl‐xL, caspase‐3, and MMP‐3, we concluded that Bcl‐xL was negatively correlated to caspase‐3 and MMP‐3 in the human body. This result, combined with previous detection results of normal and necrotic cartilage tissues, suggest that Bcl‐xL inhibits caspase‐3 and MMP‐3 in chondrocytes.

Furthermore, we determined the expression levels of cytokines IL‐1β, IGF‐1, CKLF1, and BMP in normal and necrotic cartilage tissues. The expression levels of IL‐1 β and CKLF1 were significantly higher but those of IGF‐1 and BMP were lower in necrotic cartilage tissues than in normal cartilage tissues. IL‐1β is one of the most important proinflammatory cytokines involved in the pathophysiology of osteoarthritis because it inhibits the synthesis of key components of cartilage—type II collagen and cartilage aggrecan—leading to increased cartilage degradation and aggravated inflammation [24]. IGF‐1 is a proliferative and chondrogenic redifferentiation factor with the potential to repair and regenerate cartilage defects; its levels decrease following cartilage damage. KLF1 promotes the inflammation and deterioration of chondrocytes [25], and BMP‐2 accelerates bone repair in some orthopedic diseases, although its level is generally reduced [26]. We measured the expression levels of IL‐1β, IGF‐1, CKLF1, and BMP following Bcl‐xL knockdown. The levels of IL‐1β and CKLF1 were higher but those of IGF‐1 and BMP were lower in the Si‐Bcl‐xL group than in the NC group. Combining the results of the two experiments, we found that Bcl‐xL promotes the repair and growth of chondrocytes and inhibits the deterioration caused by inflammation. Bcl‐xL gene belongs to anti‐apoptotic members, and has anti‐apoptotic activity through Bcl‐2 dependent and independent pathways [11]. Bcl‐xL could inhibit multiple factors induced apoptosis. In addition to the anti‐apoptotic effect, Bcl‐xL also has anti‐inflammatory effects and is involved in cell regeneration. Bcl‐xL lentivirus could increase the expression of Bcl‐xL protein and significantly reduce the apoptosis of chondrocytes [12]. Moreover, we detected the proliferation and apoptosis of chondrocytes after transfection. The cells in the Si‐Bcl‐xL group showed a markedly higher apoptotic rate and a notably slower growth rate than those in the NC group. Based on previous conclusions, decreased expression levels of Bcl‐xL decrease the levels of IGF‐1 and BMP, which in turn delay cell proliferation, thereby slowing down the repair of cartilage tissues. Previously study have confirmed that Genetically engineered Bcl‐xL expression in cells using a non‐viral vector could be an effective strategy for increasing cell survival after cell implantation while minimizing the potential risks. Intra articular injection of anti‐apoptotic Bcl‐xL‐engineered MSCs may provide a novel and effective approach in the treatment of cartilage defects. The expression levels of caspase‐3 and MMP‐3 were elevated following the recovery of Bcl‐xL inhibition, resulting in increased apoptotic rate and increased levels of inflammatory factors, thereby aggravating cartilage damage.

In future experiments, we will perform additional studies to explore the detailed molecular mechanisms of Bcl‐xL in cartilage damage.

## 5 CONCLUSIONS

Bcl‐xL expression is reduced following articular cartilage injury, thereby promoting the proliferation and inhibiting the apoptosis of chondrocytes. In clinical practice, Bcl‐xL can be used as a relevant molecular target indicator of osteoarthritis and other diseases that cause cartilage damage.

## CONFLICT OF INTEREST

The autors have declared no conflict of interest.

## Data Availability

The data used to support the findings of this study are available from the corresponding author upon request.
